# Preparation of Curcumin-Loaded Liposomes and Evaluation of Their Skin Permeation and Pharmacodynamics 

**DOI:** 10.3390/molecules17055972

**Published:** 2012-05-18

**Authors:** Yan Chen, Qingqing Wu, Zhenghai Zhang, Ling Yuan, Xuan Liu, Lei Zhou

**Affiliations:** Key Laboratory of New Drug Delivery Systems of Chinese Meteria Medical, Jiangsu Provincial Academy of Chinese Medicine, Nanjing 210028, China; Email: wqq18314@126.com (Q.W.); davidpharm@yeah.net (Z.Z.); yuanl.china@sina.com (L.Y.); liu_xuan1987@163.com (X.L.); youleiwuheng@yahoo.com.cn (L.Z.)

**Keywords:** curcumin, phospholipids, liposomes, skin penetration, antimelanoma activity

## Abstract

This study aimed to investigate the *in vitro* skin permeation and *in vivo* antineoplastic effect of curcumin by using liposomes as the transdermal drug-delivery system. Soybean phospholipids (SPC), egg yolk phospholipids (EPC), and hydrogenated soybean phospholipids (HSPC) were selected for the preparation of different kinds of phospholipids composed of curcumin-loaded liposomes: C-SPC-L (curcumin-loaded SPC liposomes), C-EPC-L (curcumin-loaded EPC liposomes), and C-HSPC-L (curcumin-loaded HSPC liposomes). The physical properties of different lipsomes were investigated as follows: photon correlation spectroscopy revealed that the average particle sizes of the three types of curcumin-loaded liposomes were 82.37 ± 2.19 nm (C-SPC-L), 83.13 ± 4.89 nm (C-EPC-L), and 92.42 ± 4.56 nm (C-HSPC-L), respectively. The encapsulation efficiency values were found to be 82.32 ± 3.91%, 81.59 ± 2.38%, and 80.77 ± 4.12%, respectively. An *in vitro* skin penetration study indicated that C-SPC-L most significantly promoted drug permeation and deposition followed by C-EPC-L, C-HSPC-L, and curcumin solution. Moreover, C-SPC-L displayed the greatest ability of all loaded liposomes to inhibit the growth of B16BL6 melanoma cells. Therefore, the C-SPC-L were chosen for further pharmacodynamic evaluation. A significant effect on antimelanoma activity was observed with C-SPC-L, as compared to treatment with curcumin solution *in vivo*. These results suggest that C-SPC-L would be a promising transdermal carrier for curcumin in cancer treatment.

## 1. Introduction

**Figure 1:**
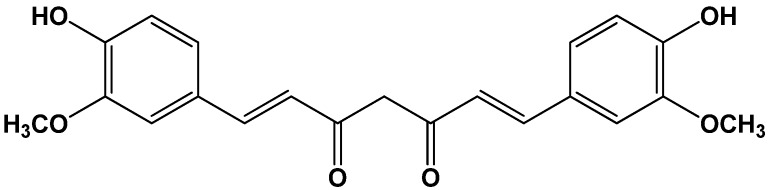
Chemical structure of curcumin.

Curcumin (Figure 1) is a compound isolated from the turmeric plant and primarily used as a natural yellow pigment. It has a variety of biological activities and pharmacological actions, such as anti-inflammatory, anti-carcinogenic, and anti-virus properties, as well as promising clinical applications due to its low toxicity [1,2,3]. In recent years, curcumin has been shown to inhibit cell proliferation in a variety of human cancer-cell lines *in vitro* [4,5,6] and has been used both to prevent and treat various cancers *in vivo* [7,8]. However, its extremely low aqueous-solubility and rapid intestinal and hepatic metabolism, which result in poor systemic bioavailability, restrict its oral use [9]. By contrast, transdermal drug delivery has many advantages over other administration routes, for example avoidance of gastrointestinal and hepatic metabolism, convenient administration for the patient, and easy withdrawal of treatment if necessary. Therefore, it has promise as a suitable administration route for curcumin. However, the highly hydrophobic properties and the excellent barrier function of the skin lead to a very low percutaneous penetration of curcumin, which makes developing a transdermal-delivery system for curcumin a challenge.

Providing a potential vehicle are liposomes, small artificial vesicles of spherical shape with a membrane composed of phospholipid bilayers [10]. They are widely used as carriers, especially in their application to topical delivery for a variety of drugs, because of their small size, biodegradability, hydrophobic and hydrophilic character, and low toxicity [11]. Results from several studies demonstrate that liposomes have the potential to enhance drug penetration into the skin, improve therapeutic effectiveness, reduce serious side effects, and act as local depots for the sustained release of drugs [12,13]. Many researchers have further reported that adding ethanol, edge activators, and some surfactant can influence the percutaneous permeability behavior of liposomes [14,15]. However, as the main component of liposomes, phospholipids, specifically, the kind of phospholipid, can directly influence the physical properties of liposomes, even their permeability behavior. 

In order to take advantage of these properties, our study aimed to develop a curcumin-loaded liposome system, which could enhance the skin delivery of curcumin. In order to investigate the influence of different phospholipids types on skin delivery of curcumin and gain access to the optimal formulation, natural phospholipids from different sources, SPC and EPC, and the synthetic phospholipid HSPC were chosen to prepare curcumin-loaded liposomes. The effect of these liposomes on *in vitro* skin permeation and antineoplastic activity was investigated by a Franz diffusion cell and B16BL6 melanoma cells cytotoxicity experiment. Moreover, the C-SPC-L that showed higher skin permeation and *in vitro* antineoplastic activity compared with the others were selected to evaluate the *in vivo* antineoplastic capacity using the melanoma-bearing mouse model.

## 2. Results and Discussion

### 2.1. Physical Characterization of Liposome Dispersions

**Figure 2:**
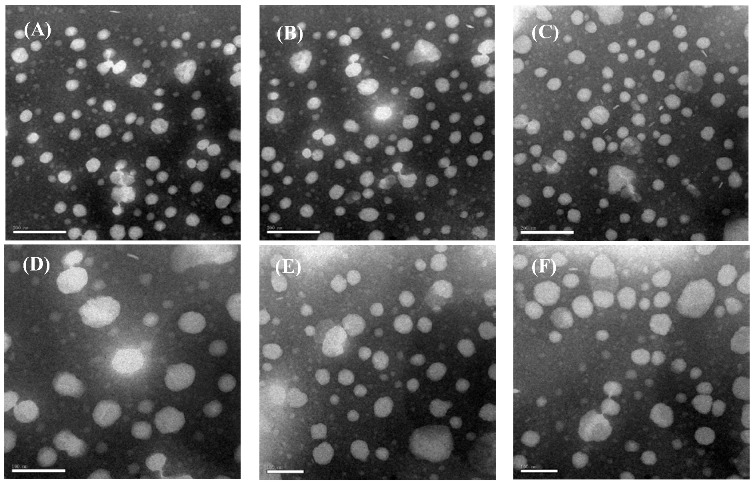
Morphology of curcumin-loaded liposomes. (**A**) C-SPC-L; (**B**) C-EPC-L; and (**C**) C-HSPC-L. Scale length: 200 nm; (**D**) C-SPC-L (2 months later); (**E**) C-EPC-L (2 months later); and (**F**) C-HSPC-L (2 months later). Scale length: 100 nm.

Particle size analysis showed that the sizes of C-SPC-L, C-EPC-L and C-HSPC-L were in the range of 82.37 ± 2.19 nm to 92.42 ± 4.56 nm (Table 1), indicating that these vesicles were all of a small size. This is a highly desirable property in terms of their topical application, since it has been shown that decreasing the vesicles’ particle size increases the penetration of encapsulated drugs into the deeper skin layers [16]. Furthermore, there was no significant difference in particle size between the C-SPC-L and C-EPC-L, whereas the particle size of the C-HSPC-L was slightly larger than that of the other two liposome types. The polydispersity index (PDI) of the investigated vesicles showed values from 0.247 ± 0.028 to 0.279 ± 0.039, indicating homogenous populations (PDI < 0.3) of vesicles (Table 1). Regarding zeta potential, all vesicles displayed a negative surface charge ranging from −10.39 ± 2.67 to −12.88 ± 1.38 mv. In general, nanoparticles could form a stable dispersion when the absolute value of zeta potential was above 30 mv due to the electric repulsion between particles. Although the absolute value of zeta potential of these liposomes were less than 30 mv, the results of the stability tests showed that the liposomes were stable at 4 °C within 2 months (see Figure 2). In addition, as shown in Table 1, the great EE values of these liposomes ranged from 80.77 ± 4.12% to 82.32 ± 3.91%, which indicates that there was no significant difference in encapsulation efficiency among these liposomes. 

**Table 1:** Characterization parameters of different formulations (mean ± SD, n = 3).

Liposome dispersion	Particle size (nm)	Polydispersity index	Zeta potential (mv)	EE (%,w/w)
C-SPC-L	82.37 ± 2.19	0.247 ± 0.028	−12.88 ± 1.38	82.32 ± 3.91
C-EPC-L	83.13 ± 4.89	0.261 ± 0.013	−11.97 ± 1.92	81.59 ± 2.38
C-HSPC-L	92.42 ± 4.56	0.279 ± 0.039	−10.39 ± 2.67	80.77 ± −4.12

### 2.2. Liposome Morphology

Transmission Electron Microscopy (TEM) was used to study the vesicles’ morphology. According to morphological evaluation analysis, all vesicle types seemed to have a similar spherical or oval shape (Figure 2). These oval-shaped vesicles may have resulted from the liposomes’ deformation, which might occur during the sample preparation. In addition, TEM failed to reveal any structural differences among these vesicle types, indicating that phospholipid type did not have a significant impact on vesicle structure.

### 2.3. Differential Scanning Calorimetry Characterization

**Figure 3:**
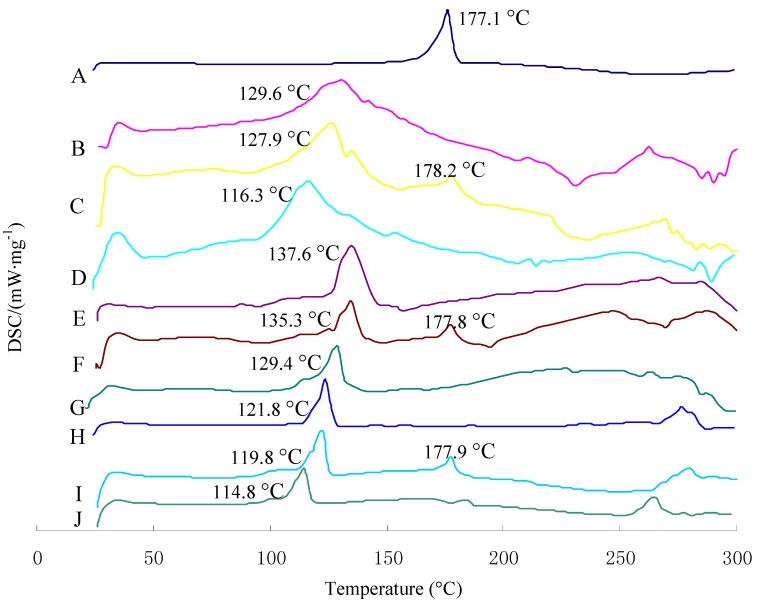
Heating curves of DSC for curcumin, physical mixture with or without curcumin, and liposomes systems: Physical mixtures I: physical mixture without curcumin; physical mixtures II: physical mixture with curcumin. A: Curcumin; B: Physical mixtures I(SPC); C: Physical mixtures II(SPC); D: C-SPC-L; E: Physical mixtures I(EPC); F: Physical mixtures II(EPC); G: C-EPC-L; H: Physical mixtures I(HSPC); I: Physical mixtures II(HSPC); J: C-HSPC-L.

Differential Scanning Calorimetry (DSC) is used to characterize the melting and crystallization behavior of crystalline materials. Figure 3 shows the DSC curves of curcumin, the physical mixture with or without curcumin, and three kinds of curcumin-loaded liposomes. The melting points for the three kinds of physical mixture I were found to be generally between 121.8 °C and 137.6 °C. Curcumin alone exhibited a melting peak of approximately 177.1 °C. The physical mixture with curcumin showed a melting point of approximately 177.1 °C; however, this phenomenon was not observed in liposomes. Moreover, the melting point of lipid material in the three kinds of liposomes dropped 5–13 °C compared with the corresponding physical mixtures I and II. These two results indicate an interaction of lipid material with the curcumin, which was in an amorphous state in the liposomes. Meanwhile, the intercalated drug molecules might disrupt hydrogen bonds spanning adjacent head-groups, thereby destroying the specific structural arrangement of a particular polar head-group region, further reducing the melting point of the liposomes’ component lipids. Similar findings were noted previously by Sainz *et al.* [17]. 

### 2.4. *In Vitro* Drug Release

**Figure 4:**
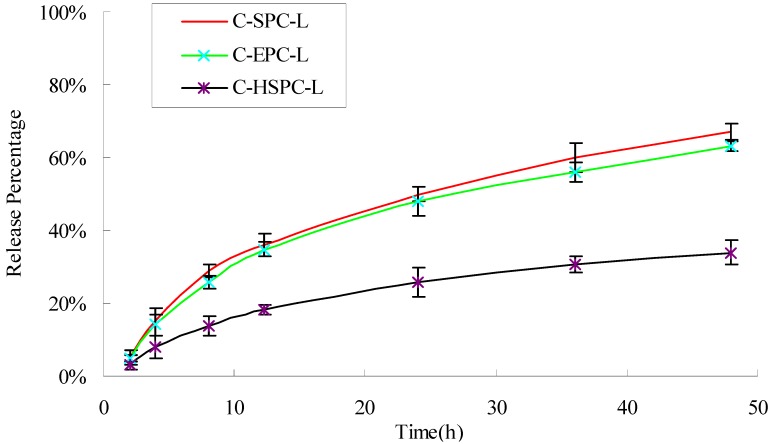
Release percentage (%) of curcumin from the C-SPC-L, S-EPC-L, and C-HSPC-L systems. Each value is represented as the mean ± S.D. (n = 3).

The *in vitro*-release profiles of curcumin obtained from the different formulations are shown in Figure 4. After 48 h, the C-SPC-L, C-EPC-L and C-HSPC-L released 67.38%, 64.22%, and 34.14% of their curcumin cargo, respectively. The C-HSPC-L clearly showed the almost one-fold lower release rate compared with that of the C-SPC-L and C-EPC-L. Similar findings were noted previously by Jun Chen *et al.* [18], who showed that the release rate of brucine encapsulated in SPC liposomes was two-fold faster than that of brucine encapsulated in HSPC liposomes. These results might be the consequence of a lower phase-transition temperature for SPC and EPC (below 0 °C) compared to that of HSPC (approximately 50 °C). The phase-transition temperature of a bilayer lipid membrane directly influences its liquidity, which in turn affects the release of curcumin from liposomes. Under the experimental temperature conditions, the lower film liquidity of the bilayer lipid membrane in C-HSPC-L was less than the C-SPC-L and C-EPC-L, which slowed down the release of curcumin from the C-HSPC-L.

### 2.5. Skin Penetration

**Figure 5:**
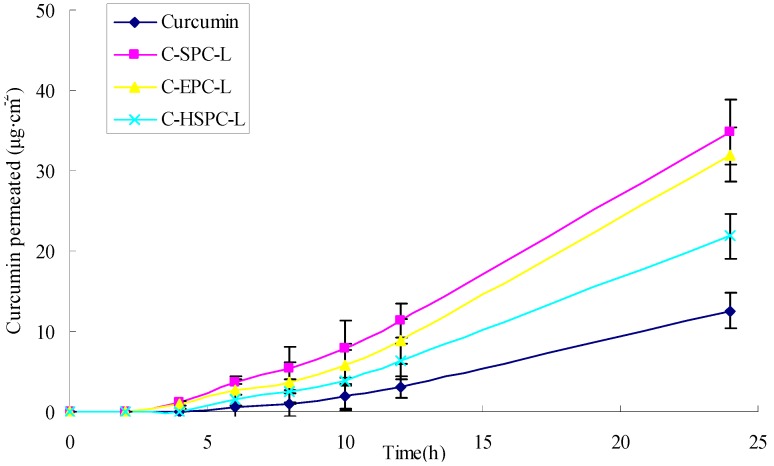
Effects of the phospholipids on curcumin permeated through excised rat skin. (mean ± SD, n = 3).

**Figure 6:**
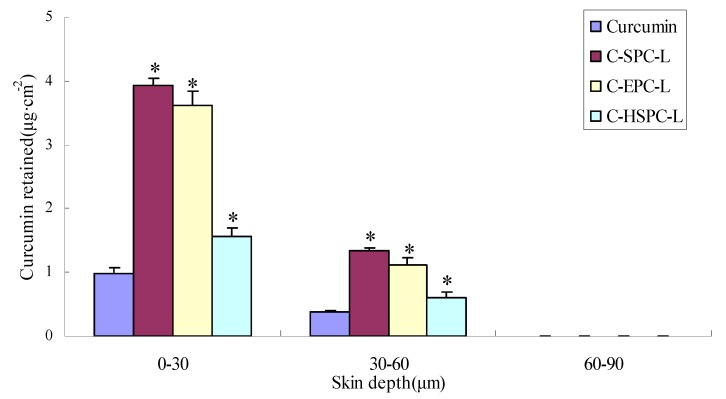
Effects of phospholipids on curcumin retained in the excised rat skin (mean ± SD, n = 3) * *p* < 0.05 versus curcumin group.

The percutaneous penetration curves and the cumulative amount of curcumin that permeated through the skin are represented in Figure 5 and Table 2, while the amounts of curcumin delivered from various formulations to different skin layers are represented in Figure 6. The cumulative amount of curcumin in different formulations that permeated through the skin in 24 h was 34.84 μg·cm^−2^ for C-SPC-L, 31.97 μg·cm^−2^ for C-EPC-L and 21.87 μg·cm^−2^ for C-HSPC-L, values which were 1.78-, 1.55- and 0.74-fold higher than curcumin solution (12.56 μg·cm^−2^), respectively. The total amount of curcumin retained in the skin showed a similar order to the cumulative amount of curcumin, which was 2.80-, 2.44-, and 1.23-fold higher than the curcumin solution. For the retention in different skin sheets, the amount of curcumin decreased in the following order: 0–30 μm > 30–60 μm > 60–90 μm (Figure 6). These results indicated the liposomes exerted a positive effect on the skin penetration and retention of curcumin. Meanwhile, the C-SPC-L and C-EPC-L provided a higher total penetration and retention amount for curcumin compared with the C-HSPC-L. This might be due to the difference of the phase-transition temperature for the different phospholipids. This temperature, which is the point at which the lipids transferred from gel to liquid phase, was directly related to the degree of the phospholipids’ unsaturation. In liquid phase, a given lipid will exchange locations with its neighbor, which permits the lipid to diffuse and thus wander across the surface of the membrane. Unlike the liquid phase, lipids in a gel phase are locked in place [19]. Therefore, at the experimental temperature conditions (37 °C), the SPC and EPC (which mainly contains unsaturated lecithin) that have lower transition temperatures, displayed better liquidity and percutaneous penetration ability compared with HSPC (hydrogenated lecithin). Similar observations were made by previously by Makiko *et al.* [20].

**Table 2:** Effects of the phospholipids on curcumin permeability and flux (mean ± SD, n = 3).

Sample	Curcumin permeated at 24 h (μg·cm^−2^)	Curcumin retained in skin at 24 h (μg·cm^−2^)	Flux (μg·cm^−2^h^−1^)
Curcumin solution	12.56 ± 2.77	1.49 ± 0.18	0.59 ± 0.11
C-SPC-L	34.84 ± 4.33	5.66 ± 0.43	1.45 ± 0.31
C-EPC-L	31.97 ± 3.37	5.23 ± 0.35	1.34 ± 0.24
C-HSPC-L	21.87 ± 2.93	3.32 ± 0.21	0.96 ± 0.18

### 2.6. Cytotoxicity

**Figure 7:**
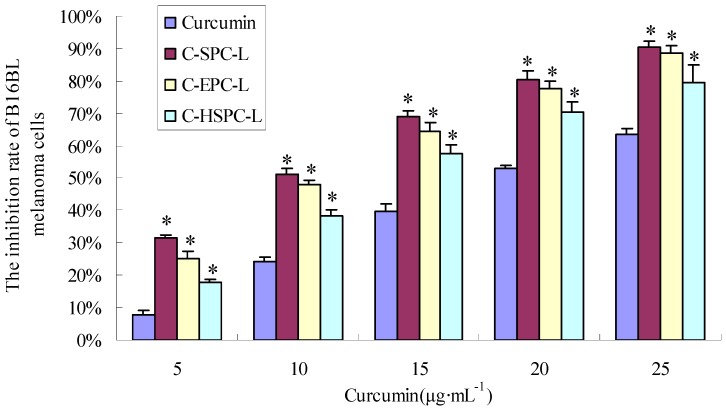
Growth-inhibiting effect of curcumin on B16BL6 cells. The results are expressed as percentage of cell-inhibition rate as compared to untreated, control cells. Data are represented as mean ± S.D. (n = 3). * *p* < 0.05 *versus* curcumin solution group.

In order to investigate the *in vitro* antitumor activity of the liposomes, they were applied to B16BL6 melanoma cells in an MTT assay. As shown in Figure 7, all curcumin formulations had a significant growth-inhibiting effect on the cell line. After treatment with curcumin liposomes and curcumin solution for 36 h, the IC_50_ values of the B16BL6 melanoma cells were 10.02 μg·mL^−1^ (C-SPC-L), 11.35 μg·mL^−1^ (C-EPC-L), 14.04 μg·mL^−1^ (C-HSPC-L) and 22.42 μg·mL^−1^ (curcumin solution), respectively. These results indicate that these liposomes can significantly increase the inhibitory effect of curcumin on B16BL6 melanoma cells. One possible mechanism for the improved antiproliferation effect could be that the fusion of the lipids’ particles and cell membrane surface promoted the transfer of drug from the extracellular to intracellular regions [21]. Phospholipids, the major components of the liposome, which have good biocompatibility could promote the delivery through the cell membrane and increase drug concentration in the cells, further enhancing the anti-cell effect of the drugs. Similar observations were made previously by Marilena *et al.* [22]. In addition, at the same concentrations of curcumin, the C-SPC-L formulation had higher cell-inhibition ratios than the other two formulations, whereas the C-HSPC-L formulation showed the lowest. There was no significant difference between C-SPC-L and C-EPC-L in cell-inhibiton ratios. At the cell-culture temperature condition (37 °C), the greater liquidity of C-SPC-L and C-EPC-L increased the ability of the liposomes to interact with the cell membrane, further promoting the release of the curcumin into the cells and enhancing its anti-proliferation effect on B16BL6 melanoma cells.

### 2.7. Effects on Tumor Growth

To assess whether curcumin-loaded liposomes foster antitumor activity *in vivo*, the C-SPC-L were chosen for pharmacodynamic evaluation. With the exception of the cyclophosphamide (positive control) group, the other groups were mixed with Carbomer hydrogels in order to obtain semisolid liposomal formulations which were convenient for application. At present, there are no marketed drugs administrated via the percutaneous route for the treatment of melanoma; therefore, cyclophosphamide, which is commonly used in antitumor experiments, was selected as the drug for positive control.

**Figure 8:**
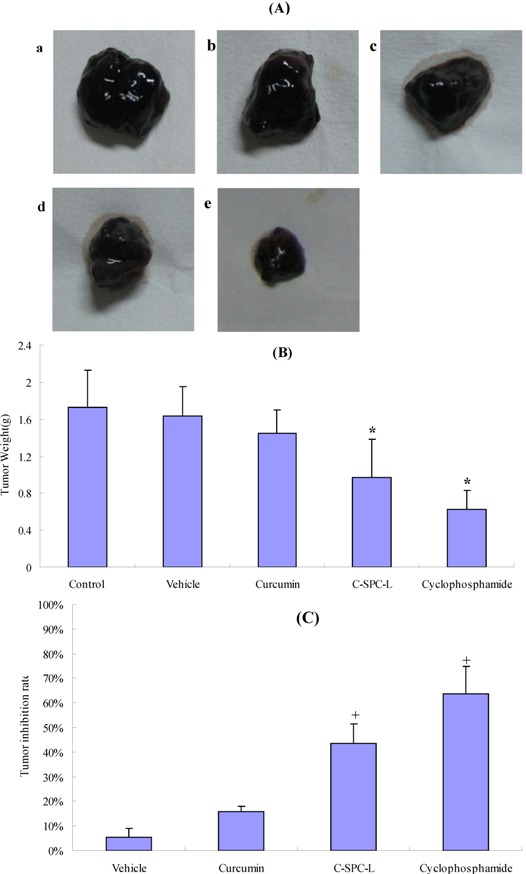
Curcumin-loaded liposomes inhibit tumor growth *in vivo*. Tumors excised from C57BL/6 mice are exhibited in (**A**). a: blank control group; b: vehicle(drug-free liposome carbomer hydrogel); c: 20 mg·kg^−1^ curcumin solution in 1% carbomer hydrogel; d: 20 mg·kg^−1^ C-SPC-L in 1% carbomer hydrogel, a-d are transdermally administered every day; e: 20 mg·kg^−1^ cyclophosphamide by intraperitoneal injection. Tumor weight and inhibition rates accounting for tumor weight are shown in (**B**) and (**C**). * *p* < 0.05 *versus* control group, + p < 0.05 *versus* vehicle group.

In Figure 8A, the tumor volume in the C-SPC-L and cyclophosphamide groups was visibly smaller than in the blank control group. The order of tumor weight of the various groups was blank control group > the vehicle group > curcumin solution group > C-SPC-L group > positive control group (Figure 8B). As can be seen from Figure 8C, the positive control and C-SPC-L group showed greater antimelanoma effects, whereas the vehicle and curcumin group exhibited only a minor effect on tumor growth.

The tumor inhibition rates of the C-SPC-L group versus the blank control group was 43.6 ± 3.6%, which was 1.74-fold higher than that of the curcumin solution group. These results indicate that the *in vivo* antimelanoma efficacy of the C-SPC-L group was significantly higher than that of curcumin alone at the same dose level of curcumin. This clearly indicates the advantage of the liposome in the delivery of curcumin.

## 3. Experimental

### 3.1. Chemicals

Curcumin (purity > 95%) was purchased from Nanjing Zelang Medical Technology Co, Ltd (Nanjing, China). SPC was provided by Shanghai Taiwei Pharmaceutical Co, Ltd (Shanghai, China). EPC and HSPC were supplied by Shanghai Advanced Vehicle Technology Co. (Shanghai, China). HPLC grade acetonitrile and acetic acid from Tedia Co. (Fairfield, OH, USA) were used for the mobile phase. Sephadex G-50 was obtained from Pharmacia (Uppsala, Sweden). All other reagents (typically analytical grade or better) were used as received. 

### 3.2. Animals and Cell Lines

Murine melanoma (B16BL6) cells were obtained from KeyGEN Biotech (Nanjing, China) and maintained in our laboratory. Male Sprague-Dawley rats (200–230 g) and male and female C57BL/6 mice (18–20 g) were obtained from the SLEK Lab Animal Center of Shanghai (Shanghai, China). The animal experiment protocol was reviewed and approved by the Institutional Animal Care and Use Committee of the Jiangsu Provincial Academy of Chinese Medicine. 

### 3.3. Liposome Preparation

Liposomes—which were composed of phospholipid, cholesterol, and Tween-80–were prepared by the conventional film method. Firstly, curcumin, phospholipid, and cholesterol were dissolved using chloroform, then the mixture was dried to a thin film at 50 °C using a rotary evaporator (ChongYe RE 3000, Shanghai, China). The obtained film was hydrated with a phosphate buffer saline (PBS) of pH 6.5 which tween-80 was dissolved in for 30 min at 60 °C. Afterwards, all liposome dispersions were sonicated (3 min, 80 W) with a probe sonicator (Noise Isolating Tamber, JY92-IIN, Ninbo, China) to obtain a small liposome particle size. SPC, EPC, and HSPC were selected as the lipids to prepare the curcumin-loaded liposomes; the cholesterol was applied to improve the liquidity of lipid membrane, and tween-80 was used to improve the encapsulation efficiency of the liposomes. The composition of the different liposome dispersions is represented in Table 3.

**Table 3:** Composition of different curcumin-loaded liposome formulations.

Formulation	Curcumin (mg)	SPC (mg)	EPC (mg)	HSPC (mg)	Cholesterol (mg)	Tween-80 (mg)	PBS pH 6.5 (mL)
C-SPC-L	2	40	-	-	5	5	1
C-EPC-L	2	-	40	-	5	5	1
C-HSPC-L	2	-	-	40	5	5	1

### 3.4. Photon Correlation Spectroscopy

The particle size and the polydispersity index (PDI) for each of these liposomes were determined by photon correlation spectroscopy (Zetasizer Nano ZS ZEN3600, Malvern Instruments Corp., Worcestershire, UK) at 25 °C under a fixed angle of 90° in polystyrene cuvettes after suitable dilution in ultrapure water. The measurements were obtained by using a 633 nm He-Ne laser. The zeta potential was measured in folded capillary cells using the Zetasizer. Liposomes were prepared by diluting with ultrapure water until the appropriate concentration of particles was achieved. The conductivity of each sample was adjusted to 50 S·cm^−1^ by 0.1 mmol·L^−1^ sodium chloride solution for zeta potential measurement. The zeta potential values were calculated using the Smoluchowski equation [23].

### 3.5. Determination of Encapsulation Efficiency

The mini-column centrifugation method was used to assay entrapment efficiency [24]. Sephadex G50 was chosen, swollen in distilled water for at least 12 h and stored at 4 °C. A small piece of cotton was inserted in the bottom of the barrels of a 2 cm^3^ injection syringe, which was then filled with sephadex G50. Excess water was centrifuged off at 1,500 rpm for 3 min, and 0.5 mL PBS (pH6.5) was added, then centrifugation repeated twice. Approximately 0.5 mL of the drug-free liposome dispersion was placed in the injection syringe, which was then centrifuged as before for presaturation. Curcumin-loaded liposomes were then added and eluted by water twice. The filtrate was accurately taken, dissolved and diluted with methanol. Following this, the drug content in the resultant solution was determined by HPLC, and the calculated drug amount was designated as W*_Entrapped_*. An equal volume of curcumin-loaded liposome suspension was also determined and the calculated drug amount was designated as W_Total_:


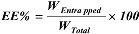


where W*_Entrapped_* and W*_Total_* were the weight of the entrapped drug and the total drug amount, respectively.

### 3.6. Transmission Electron Microscopy

The microstructures of liposomes were examined by TEM (Tecnai 12, Philips, Amsterdam, The Netherlands) using the negative-stain method. Samples were diluted appropriately with ultrapure water before preparation for TEM. A drop of each sample was applied to a copper grid coated with carbon film and the resultant construct was then air-dried. Following this, the films were negatively stained with 2% phosphotungstic acid solution and air-dried under room temperature. After these steps, the samples investigated via TEM.

### 3.7. Differential Scanning Calorimetry

DSC analysis was performed using the DSC 204 (Netzsch, Hanau, Germany). A scan rate of 10 °C·min^−1^ was employed with a temperature range of 25–350 °C. An approximately 10 mg sample was taken for analysis, with an empty pan was used as reference. Physical mixture I (containing phospholipids, cholesterol and Tween-80), physical mixture II (containing curcumin, phospholipids, cholesterol and Tween-80) and curcumin-loaded liposome samples were prepared for thermal analysis. The measurements of each sample were repeated twice.

### 3.8. *In Vitro* Drug Release

A Franz diffusion cell was used to perform the release experiment. The dialysis tubing (molecular weight cutoff of 8,000 to 14,000) was mounted between the donor and the receptor compartments. The cell provided a diffusional area of 0.785 cm^2^, and the receptor compartment was 10 mL. The donor medium consisted of 0.2 mL of vehicle containing different types of liposomes. To maintain the sink condition, 0.5% tween-80 with 20% ethanol (v/v) in pH 6.5 PBS was used as receptor medium. The system was adjusted to ensure that the membrane surface was at 37 °C to mimic the *in vivo* conditions. The stirring rate was 400 rpm and the temperature was 37 °C. At different intervals (1, 2, 4, 8, 10, 12, 18, 24, 36, and 48 h), the receptor samples were removed and replaced with fresh receptor medium. The receptor samples were then analyzed for the drug content by HPLC. The cumulative amount of drug released was determined as a function of time, and the release rate was calculated.

### 3.9. Skin Preparation

Rat abdominal skin, obtained after plastic surgery, was used for the penetration studies. The subcutaneous fatty tissue was removed from the skin using a scalpel and surgical scissors. After the fatty tissue was completely removed, the surface of the skin was cleaned with saline solution. The skin was stored in saline solution at 4 °C, then used within 1 day. 

### 3.10. Skin-Penetration Experiment

The skin permeation of curcumin was measured using a Franz diffusion assembly. The nominal surface of the Franz cell was 0.785 cm^2^ and the receptor compartments had a capacity of approximately 10 mL. The full-thickness abdominal skin was mounted between the donor and receptor compartments with the stratum corneum side facing the donor compartment. The donor medium consisted of 0.5 mL of vehicle-containing curcumin. The receptor medium had a pH of 6.5 PBS (content 0.5% tween-80 and 20% ethanol) to maintain the sink condition. The stirring rate and the temperature were kept at 400 rpm and 37 °C. At appropriate intervals (1, 2, 4, 6, 8, 10, 12, and 24 h), 1mL of receptor medium was withdrawn and immediately replaced with an equal volumes of fresh medium, The receptor samples were then analyzed for the drug content by HPLC. After the permeation experiment, the skin with the 0.785 cm^2^ permeated area was cut and washed three times using a cotton cloth containing ethanol. After this, the skin was frozen at −20 °C on a metal sample holder and sectioned three slices from the surface into 90 μm thick layers with a cryotome (Thermo-Shandon, 620 Electronic, Pittsburgh, PA, USA). The skin sheets were put together in the following scheme: vial 1 = 0–30 μm skin cuts, vial 2 = 30–60 μm skin cuts, vial 3 = 60–90 μm skin cuts. 

### 3.11. HPLC Assay

Methanol was used for the extraction of curcumin from the skin cuts. The skin cuts were extracted with 1 mL methanol and the samples were sonically extracted for 60 min. Following this, each sample was centrifuged for 15 min at 14,000 rpm (Anke TGL-16G, Shanghai, China). The supernatant was then analyzed for curcumin content by HPLC. The other samples which form the *in vitro* drug release and skin penetration experiment were centrifuged for 15 min at 14,000 rpm, and the supernatant was analyzed for curcumin content by HPLC. The HPLC system (Agilent 1100, Agilent Technologies, Palo Alto, CA, USA) consisted of a pump, a UV detector, and an Agilent-C18 column (5 μm, 4.6 × 150 mm). The mobile phase consisted of acetonitrile and 0.4% (v/v) acetic acid solution, in the ratio of 48:52 (v:v). The flow rate of the mobile phase was 1.0 mL·min^−1^. The column effluent was monitored at 430 nm. 

### 3.12. Data Analysis of Skin Penetration

Cumulative amounts of curcumin permeated over with time were used to calculate the transdermal drug flux, which was obtained from the slope of the regression line fitted to the linear portion of the profile. The skin flux can be experimentally determined from the following equation [25]: 


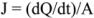


where J is the steady-state flux (μg·cm^−2^·h^−1^), A is the diffusion area of skin tissue (cm^2^) through which drug permeation takes place, and dQ/dt is the amount of drug passing through the skin per unit time at a steady-state (μg·h^−1^). 

### 3.13. MTT Assay

The MTT (3-[4,5-dimethylthiazol-2-yl]-2,5-diphenyl-tetrazolium bromide) assay was used to evaluate the cytotoxicity of curcumin and curcumin-loaded liposomes. Briefly, cells in the logarithmic growth phase were plated at a density of 0.9 × 10^4^/well in 96-well plates. 24 h later, the cells were treated with various concentrations of curcumin solution and curcumin-loaded liposomes (0, 5, 10, 15, 20, and 25 μg·mL^−1^). After incubation for 36 h, 10 μL of MTT (5 mg·mL^−1^) was added to each well, which were then incubated for 4 h at 37 °C. Following this, the supernatant was discarded and 100 μL DMSO was added to each well. The absorbance value at 550 nm was measured using a microplate reader (Thermo Labsystems, Helsinki, Finland). Any interference of the absorbance readings by particle fluorescence was monitored and accounted for. All experiments were performed with three replicates.

### 3.14. Pharmacodynamic Evaluation

Male and female C57BL/6 mice (18–20 g) of 6–8 weeks old were maintained under standard environmental conditions (temperature of 25 °C ± 2 °C and relative humidity of 50% ± 10%) and fed with a standard diet and water. The B16BL6 melanoma cells were trypsinized, resuspended in PBS (2 × 10^7^ cells/mL), and subcutaneously injected into the notum of the C57BL/6 mice. The mice were randomly divided into five groups (*n* = 10/group) according to the treatment received: The blank control, curcumin solution, vehicle, C-SPC-L and cyclophosphamide. Cyclophosphamide (20 mg·kg^−1^) was administered every day by intraperitoneal injection (i.p.) as the positive control. The other treatments were administered by percutaneous penetration daily. For curcumin-based treatments, 20 mg·kg^−1^ of curcumin in 1% carbomer gel was used. After 16 consecutive days, the tumors were excised and weighed [26].

### 3.15. Statistical Analysis

Data are mean ± standard deviation (S.D.) from three independently performed experiments. Statistical analysis was carried out using the One-way ANOVA (SPSS 11.5 software). *p* < 0.05 was considered statistically significant.

## 4. Conclusions

In conclusion, different types of phospholipids can directly influence the penetration behavior of liposomes. Although all the liposomes we constructed in this experiment offered the significant improvement in the skin penetration, deposition, and antimelanoma activity of curcumin, the C-SPC-L formulation was most effective. The aforementioned results suggest that C-SPC-L could be a promising transdermal carrier for curcumin in the treatment of skin cancer.

## References

[1] Shang Y.J., Jin X.L., Shang X.L., Tang J.J., Liu G.Y., Dai F., Qian Y.P., Fan G.J., Liu Q., Zhou B. (2010). Antioxidant capacity of curcumin-directed analogues: Structure-activity relationship and influence of microenvironment. Food Chem..

[2] Lee K.H., Aziz F.H.A., Syahida A., Abas F., Shaari K., Israf D.A., Lajis N.H. (2009). Synthesis and biological evaluation of curcumin-like diarylpentanoid analogues for anti-inflammatory, antioxidant and anti-tyrosinase activities. Eur. J. Med. Chem..

[3] Tang H.D., Murphy C.J., Zhang B., Shen Y.Q., van Kirk E.A., Murdoch W.J., Radosz M. (2010). Curcumin polymers as anticancer conjugates. Biomaterials.

[4] Hua W.F., Fu Y.S., Liao Y.J., Xia W.J., Chen Y.C., Zeng Y.X., Kung H.F., Xie D. (2010). Curcumin induces down-regulation of EZH2 expression through the MAPK pathway in MDA-MB-435 human breast cancer cells. Eur. J. Pharmacol..

[5] Lekha Nair K., Thulasidasan A.K.T., Deepa G., Anto R.J., Vinod Kumar G.S. (2012). Purely aqueous PLGA nanoparticulate formulations of curcumin exhibit enhanced anticancer activity with dependence on the combination of the carrier original. Int. J. Pharm..

[6] Wena Y.D., Ho Y.L., Shiau R.J., Yeh J.K., Wua J.Y., Wanga W.L., Chiou S.J. (2010). Synergistic antitumor effect of curcumin and dinitrosyl iron complexes for against melanoma cells. J. Organomet. Chem..

[7] Dhillon N., Aggarwal B.B., Newman R.A., Wolff R.A., Kunnumakkara A.B., Abbruzzese J.L., Ng C.S., Badmaev V., Kurzrock R. (2008). Phase II trial of curcumin in patients with advanced. Clin. Cancer Res..

[8] Dhillon N., Wolff R.A., Abbruzzese J.L., Hong D.S., Camachi L.H., Li L. (2006). Phase II clinical trial of curcumin in patients with advanced pancreatic cancer. J. Clin. Oncol..

[9] Shi S., Manjul M. (2010). Comparative bioavailability of curcumin, turmeric and Biocurcumax^TM^ in traditional vehicles using non-everted rat intestinal sac model. J. Funct. Foods.

[10] Yang F., Jin C., Jiang Y., Li J., Di Y., Ni Q., Fu D. (2011). Liposome based delivery systems in pancreatic cancer treatment: From bench to bedside. Cancer Treat. Rev..

[11] Muthu M.S., Singh S. (2009). Targeted nanomedicines: Effective treatment and brain disorders. Nanomedicine.

[12] Seth A.K., Misra A., Umrigar D. (2004). Topical liposomal gel of idoxuridine for the treatment of herpes simplex: Pharmaceutical and clinical implications. Pharm. Dev. Technol..

[13] Mura P., Maestrelli F., Gonzalez-Rodrıguez M.L., Michelacci I., Ghelardini C., Rabasco A.M. (2007). Development, characterization and *in vivo* evaluation of benzocaine-loaded liposomes. Eur. J. Pharm. Biopharm..

[14] Nina D.C., Dietrich S., Volker A., Alfred F. (2009). Development of liposomes containing ethanol for skin delivery of temoporfin: Characterization and *in vitro* penetration studies. Colloids Surf. B.

[15] El Zaafarany G.M., Awad G.A., Holayel S.M., Mortada N.D. (2010). Role of edge activators and surface charge in developing ultradeformable vesicles with enhanced skin delivery. Int. J. Pharm..

[16] Verma D.D., Verma S., Blume G., Fahr A. (2003). Particle size of liposomes influences dermal delivery of substances into skin. Int. J. Pharm..

[17] Sainz M.C., Chantres J.R., Elorza B., Elorza M.A. (1993). DSC study of action of phenylbutazone on phospholipid phase transitions. Int. J. Pharm..

[18] Chen J., Lin A.H., Chen Z.P., Wang W., Zhang T., Cai H., Cai B.C. (2010). Ammonium sulfate gradient loading of brucine into liposomes: Effect of phospholipid composition on entrapment efficiency and physicochemical properties *in vitro*. Drug Dev. Ind. Pharm..

[19] Sebastian Z., Wolfgang P., Jiirgen L. (1995). Interaction of phosphatidylcholine liposomes with the human stratum corneum. BBA Biomembranes.

[20] Makiko F.J., Kumi S., Yoichi W., Mitsuo M. (2001). Effect of phosphatidylcholine on skin permeation of indomethacin from gel prepared with liquid paraffin and hydrogenated phospholipid. J. Pharm. Pharmacol..

[21] Kien X.N., Hiroshi U., Toshinori S., Huong T.B., Ryoichi K. (2008). Enhanced release of chitosanase from streptomyces griseus through direct interaction of liposome with cell membrane under heat stress. J. Biosci. Bioeng..

[22] Marilena C., Maria G.C., Stefania B., Stefania B., Donatella P., Franco A., Domenicoantonio R., Sebastiano F., Massimo F., Diego R. (2004). Cytotoxic effects of Gemcitabine-loaded liposomes in human anaplastic thyroid carcinoma cells. BMC Cancer.

[23] Deshiikan S.R., Papadopoulos K.D. (1998). Modified booth equation for the calculation of zeta potential. Colloid Polym. Sci..

[24] El-Maghraby G.M., Williams A.C., Barry B.W. (2001). Skin delivery of 5-fluorouracil from ultradeformable and standard liposomes *in-vitro*. J. Pharm. Pharmacol..

[25] Liu C.H., Chang F.Y. (2011). Development and characterization of eucalyptol microemulsions for topic delivery of curcumin. Chem. Pharm. Bull..

[26] Song J., Shi F., Zhang Z.H., Zhu F.X., Xue J., Tan X.B., Zhang L.Y., Jia X.B. (2011). Formulation and evaluation of celastrol-loaded liposomes. Molecules.

